# *In vivo* wide-field calcium imaging of mouse thalamocortical synapses with an 8 K ultra-high-definition camera

**DOI:** 10.1038/s41598-018-26566-3

**Published:** 2018-05-29

**Authors:** Eriko Yoshida, Shin-Ichiro Terada, Yasuyo H. Tanaka, Kenta Kobayashi, Masamichi Ohkura, Junichi Nakai, Masanori Matsuzaki

**Affiliations:** 1 Department of Physiology, Graduate School of Medicine, The University of Tokyo, Tokyo, Japan; 2 Section of Viral Vector Development, National Institute for Physiological Sciences, Aichi, Japan; 3 Brain and Body System Science Institute, Saitama University, Saitama, Japan; 4 International Research Center for Neurointelligence (WPI-IRCN), The University of Tokyo Institutes for Advanced Study, Tokyo, 113-0033 Japan

## Abstract

*In vivo* wide-field imaging of neural activity with a high spatio-temporal resolution is a challenge in modern neuroscience. Although two-photon imaging is very powerful, high-speed imaging of the activity of individual synapses is mostly limited to a field of approximately 200 µm on a side. Wide-field one-photon epifluorescence imaging can reveal neuronal activity over a field of ≥1 mm^2^ at a high speed, but is not able to resolve a single synapse. Here, to achieve a high spatio-temporal resolution, we combine an 8 K ultra-high-definition camera with spinning-disk one-photon confocal microscopy. This combination allowed us to image a 1 mm^2^ field with a pixel resolution of 0.21 µm at 60 fps. When we imaged motor cortical layer 1 in a behaving head-restrained mouse, calcium transients were detected in presynaptic boutons of thalamocortical axons sparsely labeled with GCaMP6s, although their density was lower than when two-photon imaging was used. The effects of out-of-focus fluorescence changes on calcium transients in individual boutons appeared minimal. Axonal boutons with highly correlated activity were detected over the 1 mm^2^ field, and were probably distributed on multiple axonal arbors originating from the same thalamic neuron. This new microscopy with an 8 K ultra-high-definition camera should serve to clarify the activity and plasticity of widely distributed cortical synapses.

## Introduction

To understand neocortical information processing, it is important to be able to detect the fast events of action potentials in multiple neurons in a wide (≥1 mm) cortical area, as in addition to local cortical interactions, cortico-cortical and subcortico-cortical interactions are also very important^1^. Cortico-cortical and subcortico-cortical signals are carried by long-range projecting axons^2–5^. They make contact with postsynaptic sites via axonal boutons, a tiny structure of ≤1 µm^6,7^, which is much smaller than the neuronal soma of 10–15 µm. The activity patterns of multiple axonal boutons determine the activity of postsynaptic neurons, and it is therefore desirable to detect axonal activities, as well as neuronal soma activities.

Two-photon laser-scanning microscopy (TPLSM) is the most common form of imaging for multiple neuronal somata in living animals; this is because of the high spatial resolution and deep imaging depth (up to 1200 µm)^8–14^. In standard TPLSM, a single excitation laser, which has a pulse repetition rate of approximately 80 MHz, is two-dimensionally scanned using two mirrors. Therefore, if the frame rate is maintained around the normal video rate (30 fps), the pixel number per image is limited to ~2 M pixels. An image with 1024 × 1024 pixels (two or three laser pulses per pixel) is the upper limit, and an image with 512 × 512 pixels is usually used. Assuming that the pixel size required to resolve a single neuronal soma is ~5 µm, the imaging field may be extended to 2.5–5 mm. However, if the mean size of a single axonal bouton is assumed to be around 0.5 µm^7^, the pixel size required to resolve single synapses is ~0.2 µm; this permits a maximal image size of only 100–200 µm. This indicates that the current standard for TPLSM can resolve axonal activity in a part of each cortical area^15–17^, but that it is not suitable for wide-field (≥1 mm) high-speed imaging of multiple axonal boutons unless the excitation laser beam is multiplexed before entering the scanning mirrors to increase the imaging fields^18,19^.

For wide-field imaging, one-photon epifluorescent microscopy (OEFM) with a low-magnification objective is useful. Although OEFM has a lower spatial resolution than TPLSM and the imaging depth within the tissue is limited to 150–200 µm^20,21^, if the labeled neurons and their temporal activity are sparse, out-of-focus fluorescence changes that reflect neuronal activity do not usually cause deterioration to the changes in the fluorescence signals, and the activity of a single neuronal soma can usually be resolved *in vivo*^20–23^. However, in OEFM, light scattering within the tissue is more severe than in TPLSM, and single axonal boutons cannot be resolved.

**Figure 1:**
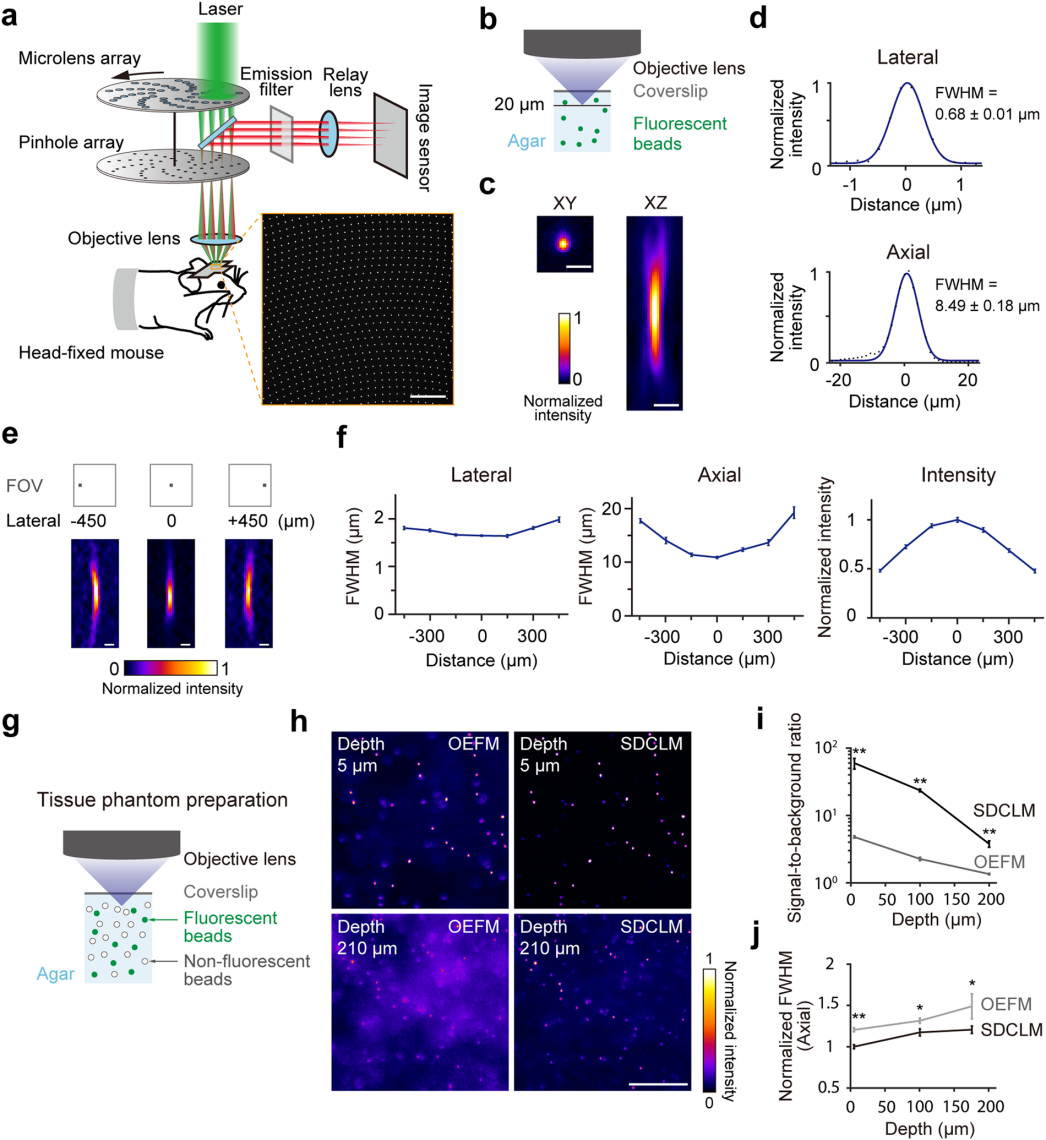
Optical performance of SDCLM. (**a**) Schematic principle of SDCLM. Arrayed microlenses split the excitation laser beam (green) and focus each of the split beams into its corresponding pinhole. The laser beam passing the pinhole focuses through the objective lens to the corresponding spot and excites fluorophores. Emitted fluorescence (red) passes the corresponding pinhole and focuses onto the corresponding photosensor. Each pinhole prevents out-of-focus fluorescence from reaching the corresponding photosensor. Right bottom panel, a representative image of non-rotating pinholes at the sample plane. Scale bar, 200 µm. (**b**) To measure the spatial resolution in SDCLM imaging (**c,d**), fluorescent beads were embedded in 0.5% agarose gel and Z-stacks of the beads located around a depth of 20 µm from the coverslip were acquired. (**c**) Representative normalized XY (left) and XZ (right) SDCLM images of fluorescent beads with a diameter of 0.5 µm. Scale bar, 2 µm. (**d**) The FWHMs of the 0.5 µm beads along X (top) and Z (bottom) axes (*n* = 5 for each axis). A Gaussian curve (blue) was fitted to the intensity profile of the bead along each axis. (**e**) Representative normalized XZ images of fluorescent beads with a diameter of 0.5 µm (bottom) at three locations along the X axial centerline in a 1 mm^2^ FOV (top). Scale bar, 5 µm. (**f**) Lateral and axial FWHMs and intensity of the 0.5 µm beads at six locations along the X axial centerline in the FOV (*n* = 5 beads for each point). The FWHMs at the center of the FOV were longer than those in **d**. This was probably because the pixel width of the camera image that was used to calculate the FWHMs was larger in **f** (1.05 µm at the sample) than in **d** (0.21 µm at the sample). (**g–j**) Performance of SDCLM compared with OEFM in a tissue phantom. (**g**) Tissue phantom preparation. Fluorescent beads with a diameter of 2.0 µm (green circles) were embedded in 0.5% agarose gel with non-fluorescent polystyrene beads (open circles) as a scattering agent, and were imaged with OEFM or SDCLM. Volumes of 1 × 1 × 0.3 mm were imaged at 2 µm spacing along the Z axis. (**h**) Representative images of the 2.0 µm fluorescent beads at two depths (top, 5 µm depth, and bottom, 210 µm depth) with OEFM (left) and SDCLM (right). The images were cropped (300 × 300 µm) from the original images (1 × 1 mm). Scale bar, 100 µm. (**i**) Signal-to-background ratios as a function of the imaging depth, where the signal was the maximum fluorescence intensity in a 30 × 30 µm square that had an in-focus fluorescent bead at the center and did not appear to have any other one, and the background was the average intensity of this square excluding a circle with a diameter of 10 µm at the center of the square. Data were presented as mean ± SEM. **p < 0.01, *n* = 5 beads, Wilcoxon rank sum test. (**j**) Axial resolution as a function of imaging depth. The values were normalized to the mean value at a 5 µm depth in the SDCLM image and plotted as mean ± SEM (*n* = 5 beads for each point). *p < 0.1, **p < 0.01, Wilcoxon rank sum test.

In this study, we introduce spinning-disk one-photon confocal laser microscopy (SDCLM)^24–27^. In SDCLM, an excitation laser beam and fluorescence emitted from the specimen are passed through a series of pinholes in a Nipkow disk rotating at high speed (Fig. 1a). The disk with the pinholes avoids a large amount of out-of-focus fluorescence from reaching the photodetector. As the distance between pinholes increases, the out-of-focus fluorescence signals decrease^28–30^. Thus, SDCLM has a three-dimensional resolution similar to that of one-photon confocal laser-scanning microscopy (CLSM), and its scanning speed is higher than that of CLSM. In contrast to CLSM, fluorescence signals passing through multiple pinholes are two-dimensionally projected to the face of a photodetector. To assign the fluorescence signal to individual small pixels suitable for resolving single synapses, we apply an 8 K ultra-high-definition (8 KUHD) CMOS camera, which currently has the highest pixel numbers available (7680 × 4320 pixels) and a frame rate of 60 fps. We demonstrate that SDCLM with the 8KUHD camera (8K-SDCLM) can resolve the activity of mouse thalamocortical (TC) axonal boutons in a 1 mm^2^ field of view (FOV), that it can do this at a single-bouton resolution, and that some axonal boutons at a distance of ~1 mm apart, which putatively originate from single axons, exhibit very similar activity.

## Results

### Imaging system for the SDCLM

To image a wide field, a spinning-disk device (Yokogawa, CSU-W1) was connected to a variable zoom microscope (Axio Zoom.V16; Carl Zeiss) with a 2.3 × objective (Plan-NEOFLUAR Z 2.3×/0.57; Carl Zeiss). In this study, we fixed the zoom size to image a FOV of 1 mm. The number of pinholes scanning the FOV was 1090, with the spacing between neighboring pinholes being 500 μm, which corresponded to 38.5 μm at the focal plane (Fig. 1a). The lateral full-width at half-maximum (FWHM) of 0.5 µm diameter fluorescent beads around the center of the FOV was 0.68 ± 0.01 µm (mean ± SEM, *n* = 5, fitted to a Gaussian function), and the axial FWHM was 8.49 ± 0.18 µm (Fig. 1b–d). In TPLSM, the lateral FWHM was 0.61 ± 0.00 µm (*n* = 5) and the axial FWHM was 3.16 ± 0.01 µm. Therefore, the axial FWHM in the SDCLM was 2.7-fold longer than that in TPLSM, and 1.1–2.1-fold longer than values previously reported for *in vivo* imaging with TPLSM^14,22,31,32^. The lateral and axial FWHMs and the fluorescence intensity degraded by up to ~60% towards the edge of the FOV (Fig. 1e,f), which is similar to the degradation occurring in wide-field TPLSM imaging^32^. If SDCLM more effectively rejects the out-of-focus fluorescence than OEFM, SDCLM should permit deeper penetration than OEFM in a fluorescence-scattered specimen. We confirmed this by measuring the SDCLM and OEFM fluorescence signals from 2 µm beads scattered in a light-scattering phantom gel^33^ (Fig. 1g–j). Considering that the thickness of each cortical layer in the mouse is >100 µm, we expected that, in contrast to OEFM, SDCLM would be able to detect sub-layer-specific neuronal activity, and that fluorescent changes around the focal plane would be detected with a larger dynamic range.

### *In vivo* calcium imaging of layer 2/3 neurons with SDCLM

**Figure 2:**
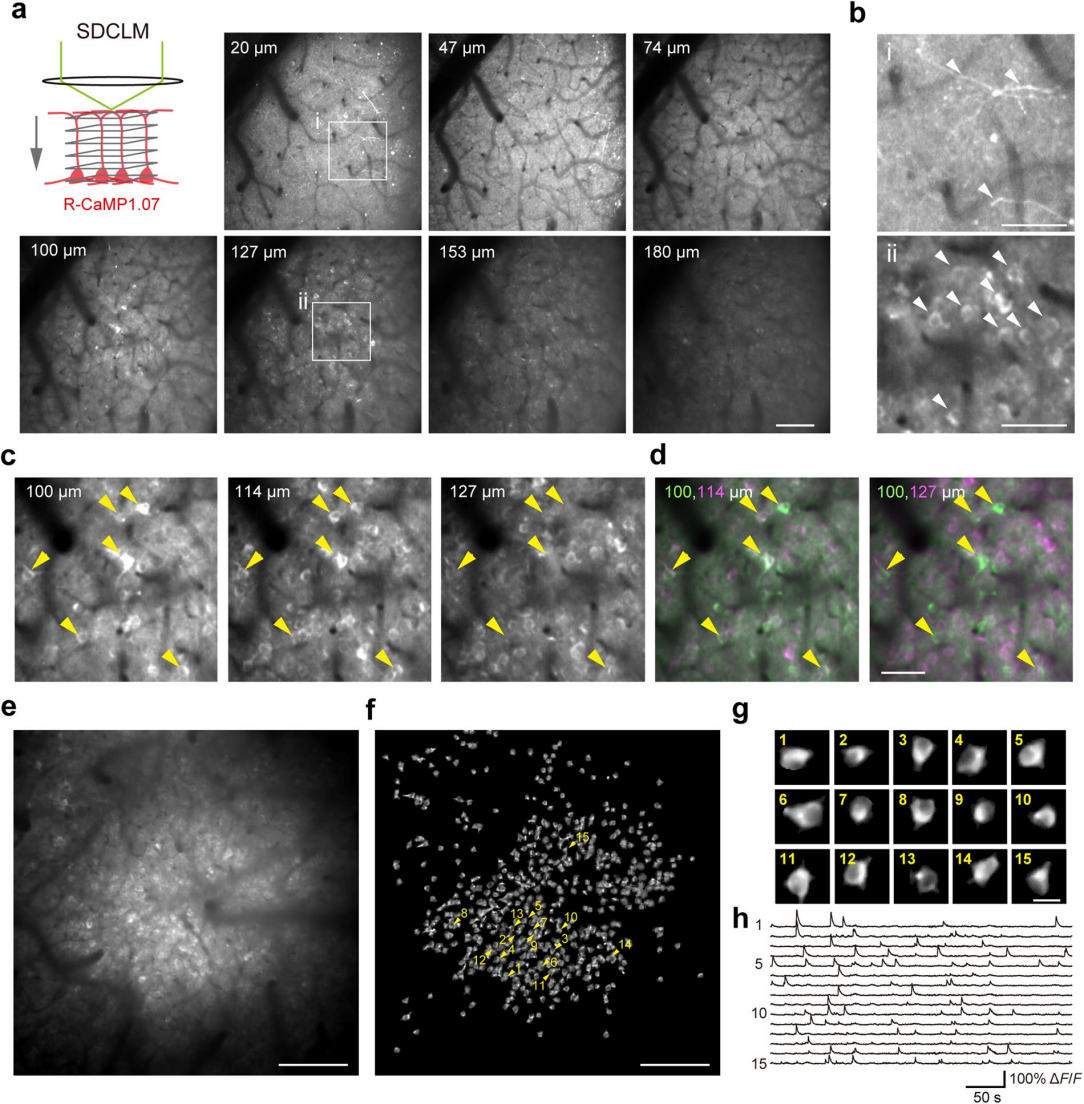
SDCLM imaging of *in vivo* R-CaMP1.07-expressing L2/3 neurons. (**a**) SDCLM imaging of R-CaMP1.07-expressing neurons at seven cortical depths in the somatosensory cortex. The images were cropped (500 × 500 µm) from the original images (1 × 1 mm). Each image was the average of 4 s of imaging at 25 fps. Scale bar, 100 µm. (**b**) Magnified images from depths of 20 µm (i) and 127 µm (ii) in **a**. Arrowheads indicate putative dendrites in i and neuronal somata in ii. Scale bars, 50 µm. (**c**) Magnified images from depths of 100, 114, and 127 µm in **a**. Yellow arrowheads indicate neuronal somata observed at 100 and 114 µm, but not at 127 µm. (**d**) Composite images from **c**. The green channel corresponds to the depth of 100 µm; the magenta channel corresponds to 114 µm in the left panel and 127 µm in the right panel. Scale bar, 50 µm. (**e**) SDCLM imaging of L2/3 neuronal somata that expressed R-CaMP1.07 in the motor cortex. The depth was 120 µm from the cortical surface. The image was the average projection during the entire imaging period (10,000 frames over 500 s). Scale bar, 200 µm. (**f**) All the spatial components in image **e** are extracted by the constrained non-negative matrix factorization algorithm. (**g**) Fifteen representative extracted spatial components. Each component corresponds to the arrowheads in **f**. Scale bar, 20 µm. (**h**) Δ*F/F* traces extracted from the neuronal somata corresponding to ROIs in **g**.

To examine whether SDCLM could detect neuronal activity at a sub-layer resolution, we applied SDCLM to calcium imaging of neuronal somata in the mouse dorsal neocortex. We used a monochromic EM-CCD camera with 1024 × 1024 pixels (iXon Ultra 888; Andor Technology) as a photodetector. The red-shifted fluorescent genetically encoded calcium indicator (red GECI) R-CaMP1.07 was expressed in the somatosensory cortical neurons following adeno-associated virus (AAV)-based transfection. A cranial window was made above the injection sites for optical access. Four weeks after the AAV injection and the cranial window setting, we imaged the cortex in the awake head-fixed mouse. To compare the structural differences through cortical layers, we acquired volumetric images from 0 to 190 µm below the cortical surface (Fig. 2a). SDCLM captured subcellular morphology at depths of 20–74 µm below the cortical surface, within layer 1 (L1; depths of 0–100 µm), where there were few pyramidal neurons (Fig. 2bi). At depths of 100–153 µm, which were within layer 2/3 (L2/3; depths of 100–250 µm), many neuronal somata were observed (Fig. 2bii). However, imaging at a depth of 180 µm and below was blurred, and the fluorescent structures of the neuronal soma were unclear. Notably, L2/3 neuronal somata observed at a particular focal plane mostly disappeared when the focal plane was moved 27 µm deeper (Fig. 2c,d). This is rational for imaging of neuronal soma with a diameter of 10–15 µm. Thus, SDCLM was demonstrated to have a sub-layer axial resolution for depths down to ~180 µm from the cortical surface.

We then acquired a time series of fluorescence signals from the motor cortex, at a cortical depth of 120 µm and a rate of 20 fps (Fig. 2e, Supplementary Video 1). Neuronal somata with calcium transients were extracted using a constrained non-negative matrix factorization (CNMF) algorithm^34^. The algorithm identified 558 putative neuronal somata from the whole FOV (Fig. 2f). The extracted regions of interest (ROIs) showed a ring shaped structure, reflecting nuclear excluded expression of R-CaMP1.07 (Fig. 2g). This ring shape was difficult to detect with OEFM and is regarded as a feature of GECI-expressing neurons detected with TPLSM^32,35^. Some neurons showed ~100% relative fluorescence changes (Δ*F/F*) (Fig. 2h). The maximal amplitude of Δ*F/F* in individual neurons was 27.7 ± 0.6% (mean ± SEM; range from 11% to 105%), which was much higher than obtained with OEFM (~1–10%)^21,35^. These results indicate that SDCLM effectively rejected the out-of-focus fluorescence signals, and that the dynamic range of the fluorescent changes was much larger than in OEFM.

### Imaging of TC axonal boutons with 8K-SDCLM

**Figure 3:**
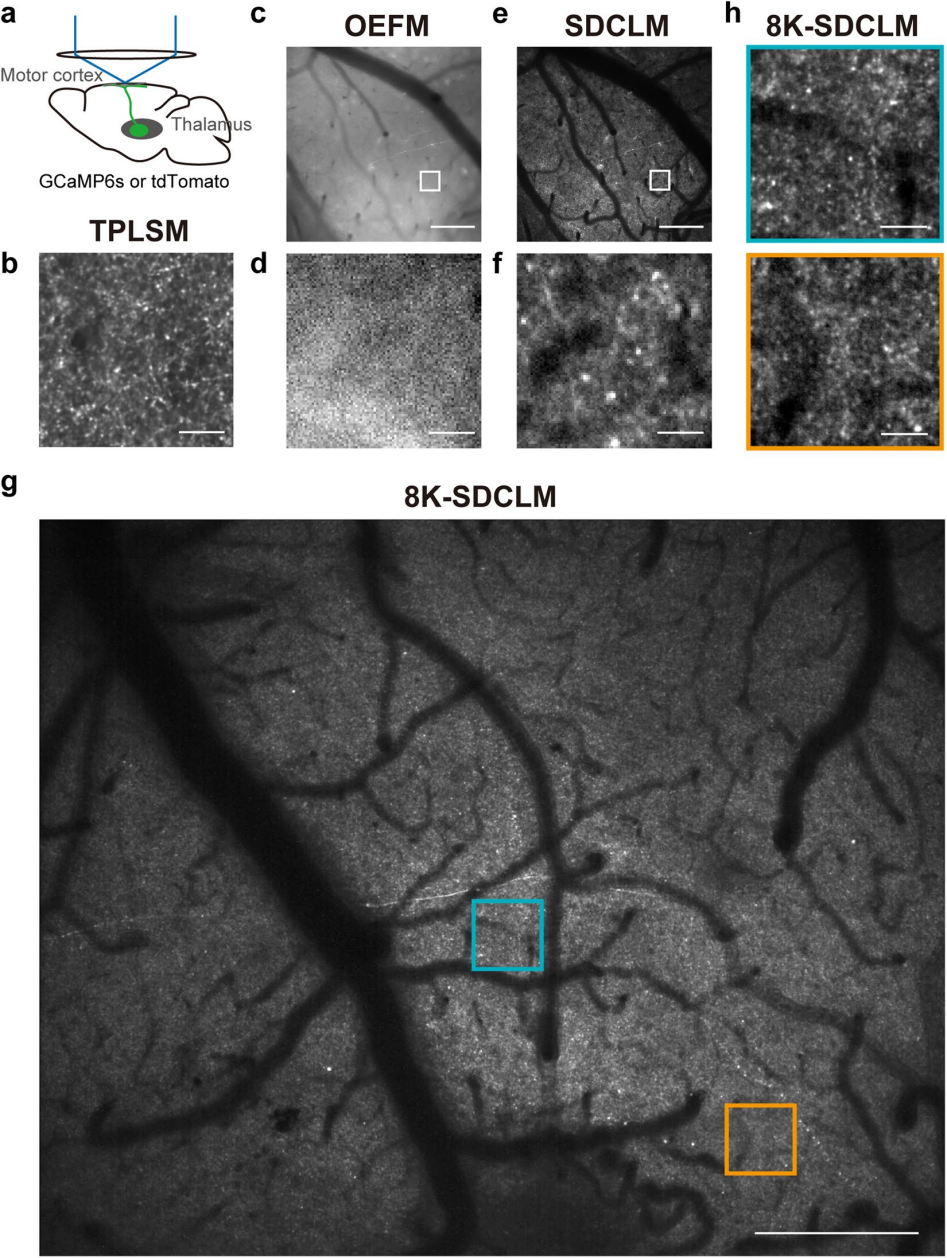
*In vivo* imaging of thalamocortical axonal boutons with TPLSM, OEFM, SDCLM, and 8K-SDCLM. (**a**) Schematic illustration of imaging of TC axons in L1. (**b**) Representative two-photon image of TC axons that expressed GCaMP6s. The image is the average projection during the imaging. The image was cropped (85 × 85 µm) from the original 127 × 127 µm acquisition. Scale bar, 20 µm. (**c**,**e**) Representative OEFM (**c**) and SDCLM (**e**) imaging of tdTomato-expressing TC axons in the same cortical field. Scale bars, 200 µm. (**d**,**f**) Magnified images of white boxes in **c** (**d**) and **e** (**f**). Scale bars, 20 µm. (**g**) 8K-SDCLM imaging of a TC axon that expressed GCaMP6s. The image is the mean projection of frames in which the frame-averaged fluorescence exceeded its time-average value plus 2S.D. Scale bar, 200 µm. (**h**) Magnified images of blue and orange boxes in **g**. Scale bars, 20 µm.

Next, we imaged TC axons in the L1 motor cortex of an awake head-fixed mouse (Fig. 3a). The motor thalamus projects axons to the L1 motor cortex, and these are thought to modulate cortical activity^4,36,37^. TPLSM is the standard way to image presynaptic boutons in the local neocortical area^15,16^, and therefore we first performed two-photon imaging of GCaMP6s-expressing TC axons in a small field of 127 × 127 µm (Fig. 3b). We detected many fluorescent spots of less than 3 µm corresponding to TC axonal boutons. Next, we evaluated whether OEFM and SDCLM could be applied to imaging of axonal boutons in a FOV of 1080 × 1080 µm. As inferred from the strong out-of-focus background signals, OEFM could not detect any fluorescent spots of less than 3 µm in L1, including tdTomato-expressing TC axons (Fig. 3c,d). By contrast, in the images obtained with SDCLM, many axonal bouton-like fluorescent spots were detected in a 1 mm^2^ FOV (Fig. 3e,f). However, the fluorescent spots appeared to be larger and their contours dimmer than the GCaMP6s-expressing TC axons obtained with TPLSM (Fig. 3b). This deterioration may be a result of the pixel size of the camera, as well as the lower optical resolution. In the two-photon imaging, the imaging field was 512 × 512 pixels and the FOV was 127 × 127 µm, giving a corresponding pixel size of 0.25 µm. By contrast, in the SDCLM, the camera had an array of 1024 × 1024 pixels covering a FOV of 1080 × 1080 µm; thus the corresponding pixel size was 1.05 µm, which would be insufficient to image single axonal boutons, which are ~0.5 µm in size^7^.

To overcome this problem, we used an 8KUHD CMOS camera (Canon) as a photodetector for the SDCLM. Because of the size of the lens in front of the photosensors, the FOV was set to 1106 × 900 µm and projected to the photosensors with an array of 5310 × 4320 pixels on the 8KUHD camera through the SDCLM. The pixel size in the specimen thereby corresponded to 0.21 µm. Using the 8K-SDCLM, we imaged GCaMP6s-expressing TC axons within a 1 mm^2^ FOV in a head-fixed mouse performing a forelimb movement task^38^ (Fig. 3g,h, Supplementary Video 2; see Methods for details). The imaging frame rate was 60 fps and the image acquisition period was 360 s. Thus, more than 20,000 frames were acquired in this experiment. The 8KUHD camera is produced for high-definition television and has three color channels (red, green, and blue), with the color filter array on the light-receiving face of the camera being a Bayer filter mosaic. Half of the total number of pixels receive green wavelengths, a quarter of pixels receive red signals, and the other quarter receive blue signals. The values for the unsampled colors/pixels are then interpolated by a demosaicing algorithm^39^. To allow use of readily available algorithms in the following analyses, the data format was converted to 16 bit tiff (see Methods for details). After imaging, all images in a part of the FOV were motion corrected with TurboReg^40^ and filtered with a 0.2 µm Gaussian kernel. In the time-averaged images, bright fluorescent spots appeared to be smaller and their contours clearer than with the 1 K camera (Fig. 3f,h). These actually appeared to be similar to the spots in the image obtained with TPLSM (Fig. 3b,h). Thus, we concluded that the 8K-SDCLM can be used for *in vivo* calcium imaging of L1 TC axonal boutons with a subcellular resolution and with a 1 mm^2^ FOV.

### Calcium transients of single axonal boutons obtained with 8K-SDCLM

**Figure 4:**
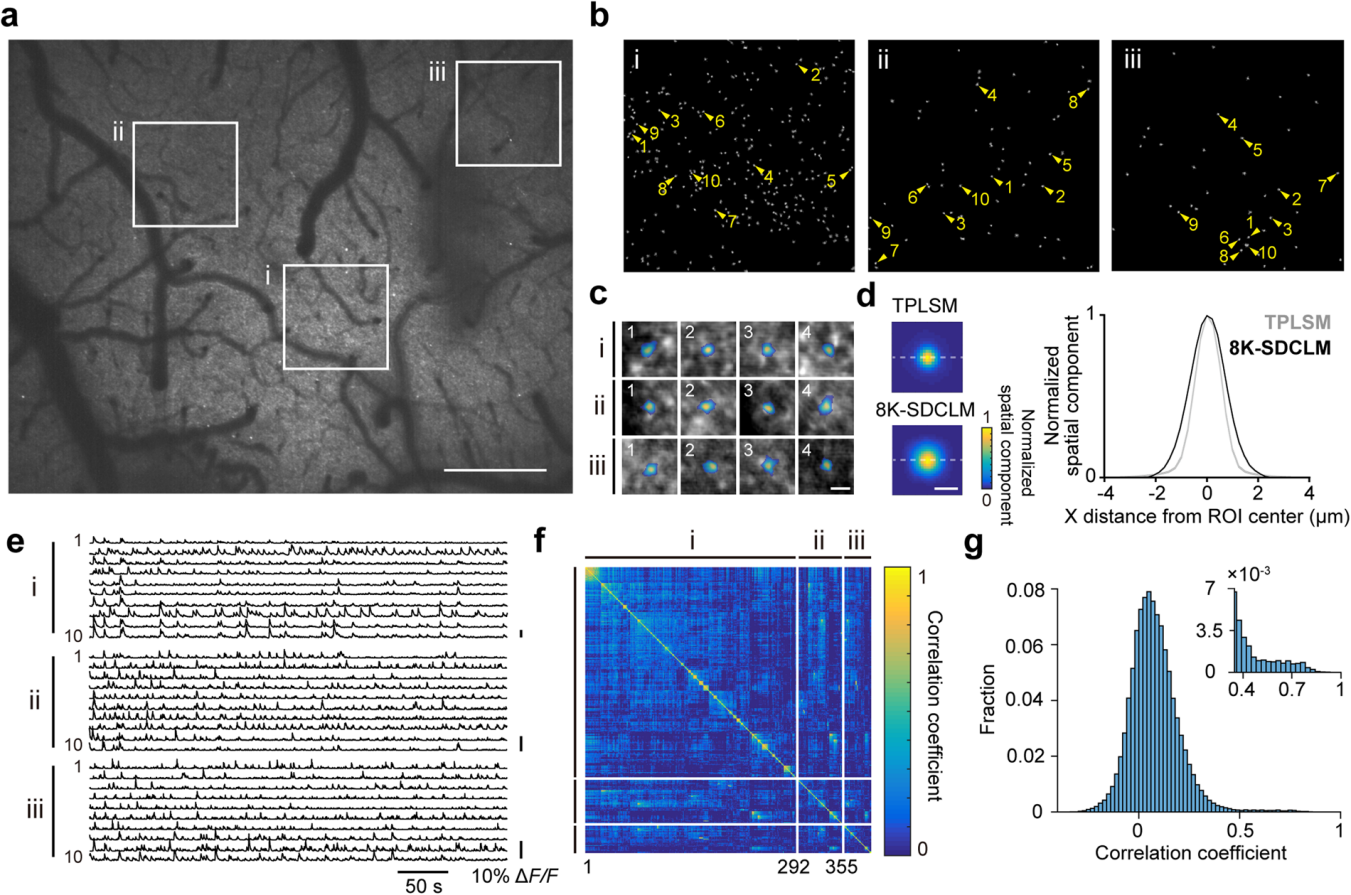
*In vivo* imaging of axonal boutons in a 1 mm^2^ cortical area. (**a**) 8K-SDCLM image of the motor cortical area overlapping the imaging field shown in Fig. 3g. The imaging experiment was performed a day after the image shown in Fig. 3g was acquired. Three areas were randomly chosen for image processing with the CNMF algorithm (i,ii,iii). The length of one side was 213 µm. The size of each square corresponded to 1024 × 1024 pixels. Scale bar, 200 µm. (**b**) All spatial components extracted by the CNMF algorithm. (**c**) Representative merged images of the pseudocolored spatial components and mean projections of highly activated frames in which the frame-averaged fluorescence exceeded its time-average value plus 3S.D. Scale bar, 4 µm. (**d**) Left, averaged images of ROIs from the images obtained by TPLSM and 8K-SDCLM (TPLSM, 1653 ROIs from six imaging fields; 8K-SDCLM, 373 ROIs from the three 213 × 213 µm areas in **a**). Individual ROIs were aligned at the center, and the normalized spatial component of each pixel was averaged and pseudocolor coded. Scale bar, 2 µm. Right, profiles of the normalized spatial components of the averaged ROIs along the dotted lines shown in the left panels. (**e**) Δ*F/F* traces extracted from 30 ROIs numbered in **b**. (**f**,**g**) Matrix (**f**) and histogram (**g**) of pair-wise correlation coefficients of the activities of ROIs in **b**. The matrix was arranged according to the orders of hierarchal clusters, on the basis of pair-wise correlation coefficients. The histogram of the correlation coefficients showed a second peak around 0.6 (inset in **g**). The bin width is 0.02 (0.03 in inset).

Next, we determined whether individual active boutons were spatially resolved when imaging with 8K-SDCLM. We randomly chose three cortical areas from the 1 mm^2^ imaging field (each area measured 213 × 213 µm), motion corrected and spatially filtered these areas (Fig. 4a), and then used the CNMF algorithm to find ROIs with calcium transients within these areas (Fig. 4b,c). A total of 395 ROIs were extracted from the three areas and defined as putative synaptic boutons; example traces of the calcium transients are shown in Fig. 4e. To compare the size of the ROIs between 8K-SDCLM and TPLSM images, we normalized the spatial components to the maximum value within each ROI (see Methods for details). When the areas in which the normalized spatial components exceeded 0.5 were defined as ROI areas, the mean diameter of the ROI areas was 1.74 ± 0.015 µm (mean ± SEM; *n* = 373 ROIs; Fig. 4d). This value was slightly higher than that from TPLSM (1.25 ± 0.0083 µm, mean ± SEM; *n* = 1653 ROIs from six imaging fields, *P* = 1.5 × 10^−152^, *t*-test). The numbers of ROIs in the areas indicated in Fig. 4bi, ii and iii were 291, 63, and 41, respectively. From these values, the number of ROIs that could be extracted from a total field of 1 mm^2^ was estimated to be 900–6400. However, the density of detected ROIs (291 per 213 × 213 µm field = 6414 ROIs/mm^2^), even around the center of the FOV, was lower than that in images from TPLSM (19,540 ± 737.0/mm^2^, mean ± SEM; *n* = 6 imaging fields).

Next, we examined whether the Δ*F/F* detected from each presynaptic bouton reflected its own activity. We used an iteratively re-weighted least-squares algorithm to remove contamination from the fluorescence laterally surrounding each ROI^41^. The maximal amplitude of the calcium transients in individual ROIs was 11.2% ± 0.3% (mean ± SEM; *n* = 395 ROIs), which was much smaller than the value from the images obtained with TPLSM (1890% ± 250%; mean ± SEM; *n* = 1891 ROIs from six imaging fields). This indicates that out-of-focus fluorescence contributed to the baseline fluorescence intensity (*F*) and lowered the amplitude of Δ*F/F* from a single bouton. If the Δ*F/F* detected from individual ROIs mostly reflected the change in locally averaged out-of-focus fluorescence, correlations between pairs of ROIs, especially pairs of adjacent ones, would be high. We therefore computed the pair-wise correlation coefficients for the fluorescent traces between each pair of ROIs (Fig. 4e,f). The median value of the calculated correlation coefficients was 0.066. This value was similar to those from the images obtained with TPLSM (from 0.031 to 0.15; *n* = 6 imaging fields). The slope of the pair-wise correlation coefficient against the distance between a pair was −1.37 × 10^−5^ µm^−1^ in 8K-SDCLM images, which was within the range of those obtained with TPLSM (from −1.60 × 10^–4^ to −2.2 × 10^−6^ µm^−1^; *n* = 6 imaging fields). These results suggest that contamination from the averaged out-of-focus fluorescence changes on the detected calcium transients was minimal. The spike event of the ROIs estimated by the CNMF algorithm was 32.7 ± 0.741 per minute (mean ± SEM; *n* = 395 ROIs). This value was less than that obtained with TPLSM (58.3 ± 0.499 per minute, mean ± SEM; *n* = 1891 ROIs from six imaging fields).

### Comparisons of ROI density and activity between 8K-SDCLM and TPLSM

**Figure 5:**
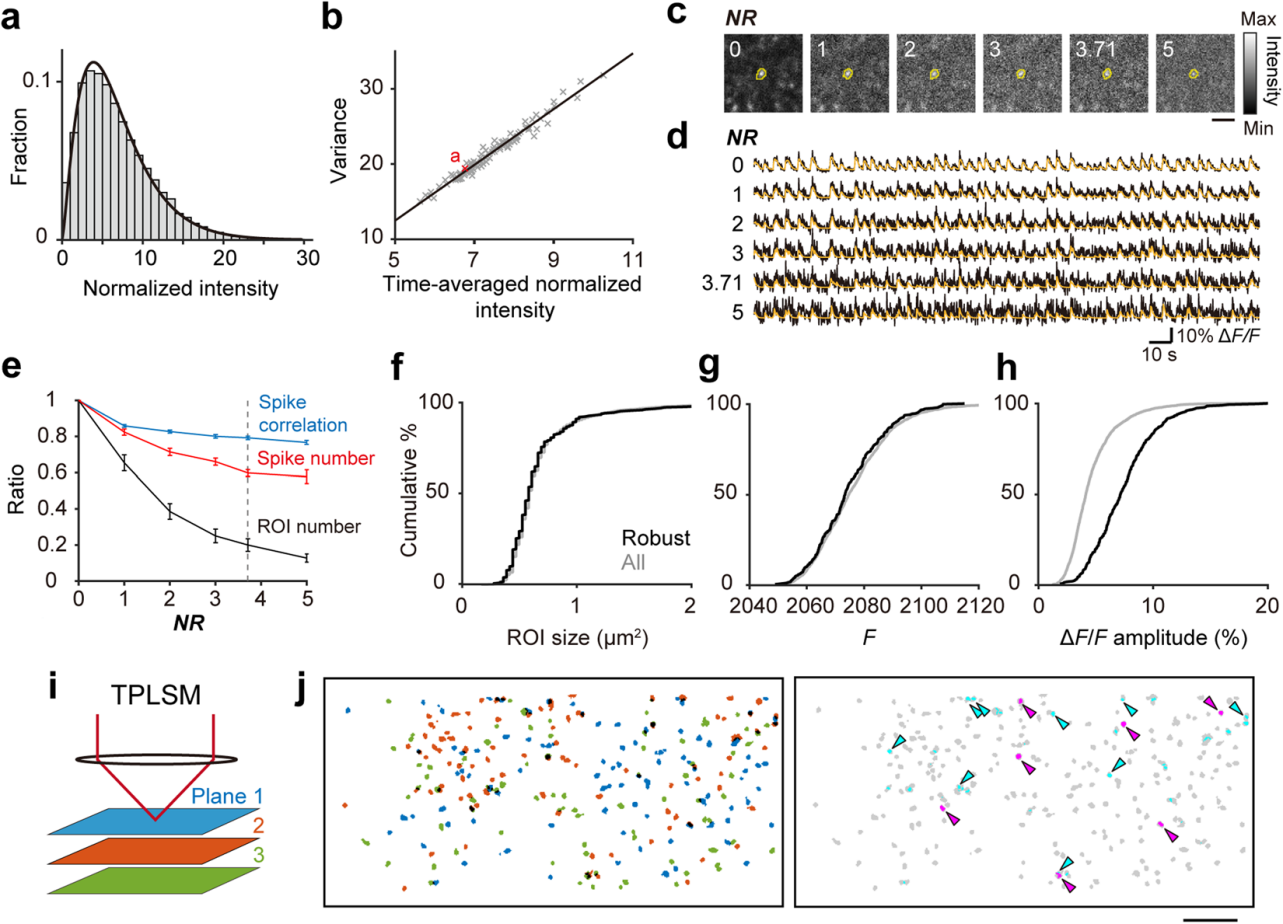
Comparisons in detected ROIs and their activity between 8K-SDCLM and TPLSM imaging. (**a**) Representative distribution of the normalized fluorescence signals from one pixel in the field shown in Fig. 4ai (21,600 time points). The fitted gamma distribution is also shown (the shape parameter is 2.35 and scale parameter is 2.87). (**b**) Relationship between the variance and time-averaged normalized fluorescence signal for 100 pixels. The red cross indicates the pixel shown in **a**. The regression line is also shown (*p* = 1.81 × 10^−75^, Pearson correlation test). (**c**) Representative 30 frame-averaged TPLSM images with the artificial noise (from left to right, *NR* = 0, 1, 2, 3, 3.71, and 5). The ROI area corresponding to the left-most one was extracted from each image and its contour was overlaid on each image. Scale bar, 5 µm. (**d**) Δ*F/F* traces of the ROI areas shown in **c**. Black traces were directly calculated from the fluorescence signals from the motion-corrected raw images with artificial noise. Orange traces were denoised traces obtained using the CNMF algorithm. (**e**) Ratios of the number of detected ROIs (black), the number of detected spike events (red), and the pair-wise correlation coefficients in the spike event between pairs of ROIs (blue) with and without the artificial noise are plotted against the added noise (*NR* = 1, 2, 3, 3.71, and 5). The dotted line indicates the *NR* of 3.71. (**f**) Cumulative distribution of the ROI area in all ROIs without the noise (gray, *n* = 1697) and the robust ROIs (black, *n* = 278). *p* = 0.797, Kolmogorov-Smirnov test. (**g**) Cumulative distribution of *F* in all ROIs without the noise (gray, *n* = 1697) and the robust ROIs (black, *n* = 278), *p* = 0.402, Kolmogorov-Smirnov test. (**h**) Cumulative distribution of the maximal amplitude of Δ*F/F* in all ROIs without the noise (gray, *n* = 1697) and the robust ROIs (black, *n* = 278). *p* = 1.84 × 10^−57^, Kolmogorov-Smirnov test. (**i**) Schematic illustration of XYZ TPLSM imaging of TC axons at three depths. (**j**) Representative spatial distribution of ROIs extracted from three 8 µm-apart fields in the same horizontal location. The left panel is the composite image of the ROIs from planes 1, (blue), 2 (orange), and 3 (green). Black areas are overlapping areas of pairs of ROIs. In the right panel, the overlapping areas of pairs of ROIs in the left panel are colored (cyan or magenta), while the ROI areas with no overlap between different planes in the left panel are colored gray. Arrowheads indicate pairs of ROIs whose overlapping area exceeded 50% of the ROI area. Magenta arrowheads indicate overlapping areas of pairs of ROIs with a pair-wise correlation coefficient in spike events of >0.6, which were removed for the calculation of the overlapping ratio (see Methods for details). Scale bar, 10 µm.

In addition to the lower mean spike events (56%), the density of detected ROIs was lower (33%) than that obtained with TPLSM as described above. If the imaging volume in the 8K-SDCLM (with an axial FWHM of ~8.5 µm) was assumed to involve 3-fold more boutons than that in TLPSM (with an axial FWHM of ~3.2 µm), the detected ROIs in 8K-SDCLM corresponded to 11% of the boutons that were detected by TPLSM. These reductions might be due to the lower signal-to-noise ratio in the 8K-SDCLM imaging. To test this possibility, we added fluorescence background noise with similar characteristics to the noise in the 8K-SDCLM images to the TPLSM images, and determined how the number of detected ROIs and their spike events decreased as the noise increased. The distribution of normalized fluorescence signals for each pixel in the 8K-SDCLM images was near to a gamma distribution (see Methods for details; Fig. 5a). The variance of the corresponding gamma distribution was linearly proportional to the time-averaged normalized fluorescence signal per pixel (Fig. 5b). This slope was defined as the noise ratio (*NR*) and had a value of 3.71 in the 8K-SDCLM data (see Methods for details). Then, a random number according to the approximated gamma distribution with the *NR* (1, 2, 3, 3.71, and 5) was added to each pixel at each time frame in the imaging data acquired with the TPLSM, and from the time series of these images, the ROIs and their spike events were detected with the CNMF algorithm (Fig. 5c,d). As the *NR* increased, the number of detected ROIs sharply dropped (black in Fig. 5e). By contrast, as the *NR* increased, the number of detected spike events decreased only slowly (red in Fig. 5e), and the correlation in spike events detected before and after the noise addition remained high, even for those ROIs detected after the noise was added (≥ ~0.8; blue in Fig. 5e). At an *NR* of 3.71, which corresponded to that in the 8K-SDCLM images, the numbers of detected ROIs and spike events were approximately 20% and 60% of those without the added noise (Fig. 5e). This simulation result was not in contradiction with the estimation from the original data and was consistent with the result in the original study describing the development of the CNMF algorithm^34^. These results indicate that although the number of boutons detected with 8K-SDCLM was 10–20% of the number detected with TPLSM, ~60% of their activity was extracted. To determine the properties of the ROIs that were extracted after addition of the noise with *NR* = 3.71 (robust ROIs), we compared the distributions of the ROI area, *F*, and maximal amplitude of Δ*F/F* at *NR* = 0 between all ROIs extracted at *NR* = 0 and the robust ROIs. The robust ROIs had similar distributions of ROI area and *F* as those extracted without the noise (Fig. 5f,g). By contrast, in the robust ROIs, the distribution of the maximal amplitude of Δ*F/F* was significantly shifted to the right side in comparison with the distribution of all ROIs (Fig. 5h). Thus, 8K-SDCLM was useful for detecting L1 TC axonal boutons with large calcium transients.

Some of the detected ROIs in the 8K-SDCLM might represent the sum of multiple active boutons located nearly perpendicular to the optical axis. If the number of ROIs detected at a 8K-SDCLM imaging plane was similar to the number of ROIs pooled from three different planes in the TPLSM imaging, spatially overlapping ROIs from these different TPLSM imaging planes might be counted as single ROIs in the 8K-SDCLM imaging. To estimate to what extent ROIs at different planes overlapped, we conducted XYZ TPLSM imaging at three depths and extracted ROIs for each plane. When three planes were overlaid, if more than 50% of one ROI area overlapped with another ROI area, the former ROI was assumed to be merged with the latter ROI. The ratio of the number of overlapping ROIs to the number of all ROIs except for the overlapping ones was only 3.81% ± 0.54% (mean ± SEM; *n* = 3 horizontal locations from three mice; Fig. 5i,j). When fluorescence signals were summed over the three planes and then Δ*F/F* in ROIs determined from each image was recalculated, the maximal amplitude of Δ*F/F* of overlapping ROIs was not different from that of ROIs with no overlapping (overlapping, 93.9% ± 3.80%, *n* = 60 ROIs; no overlapping, 89.2% ± 0.00%, *n* = 786 ROIs, mean ± SEM; *p* = 0.35, *t*-test). This suggests that the probability that a detected ROI in 8K-SDCLM consisted of multiple boutons was at most ~4%. These results demonstrate that the labeling of TC axons was sufficiently sparse for 8K-SDCLM to resolve the relatively large calcium transients of L1 TC axonal boutons.

### Multiple axonal boutons distributed over a 1 mm^2^ field might originate from the same neuron

**Figure 6:**
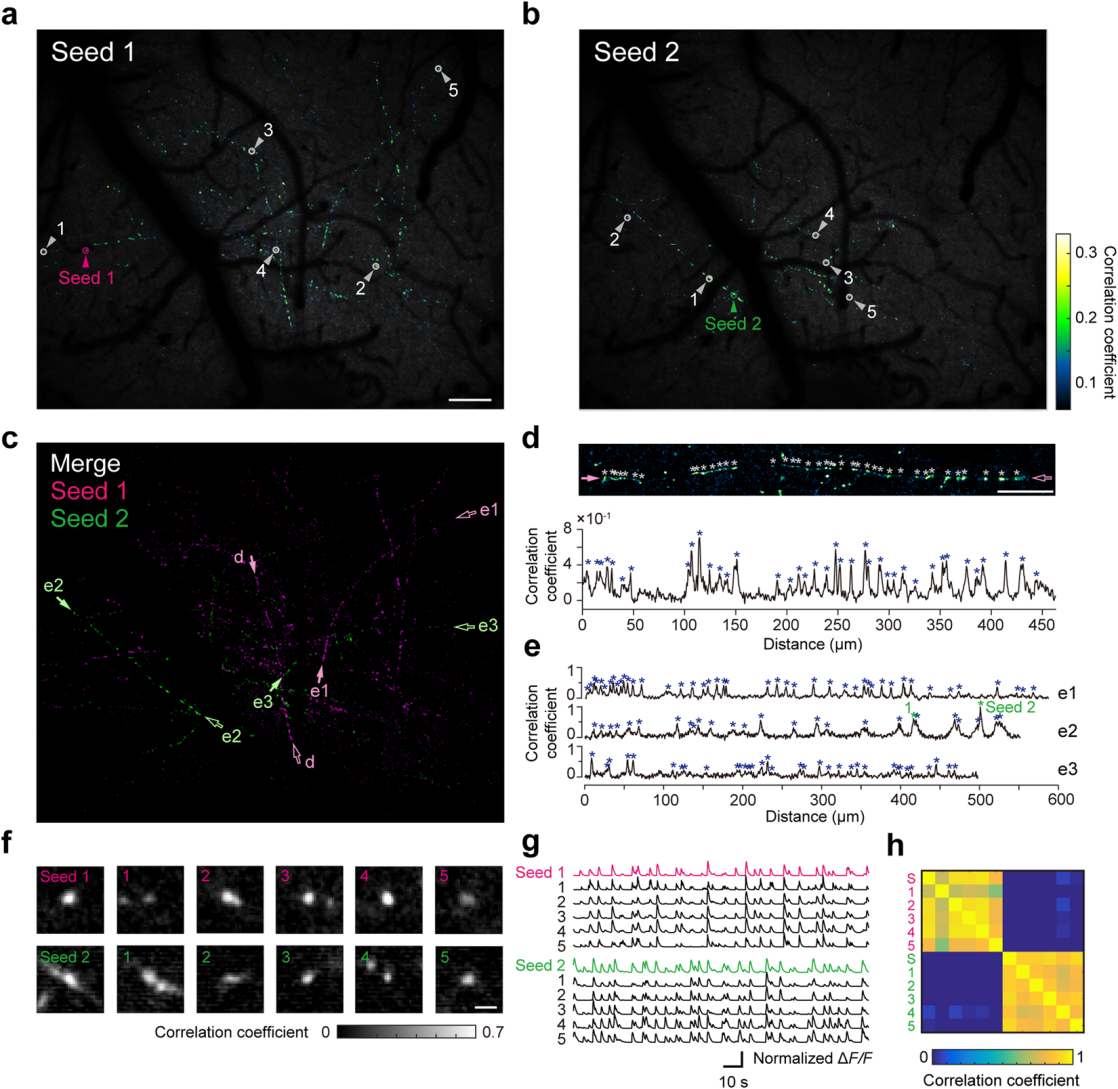
Highly correlated activity of axonal boutons on putative same axons scattered over the 1 mm^2^ cortical area. (**a**,**b**) Maps of correlation coefficients calculated by the pixel-wise correlation analyses. Magenta (**a**) and green (**b**) arrows show each seed. Scale bar, 100 µm. (**c**) Composite image of **a** and **b**, indicating no overlap. (**d**) Top, magnified image of a putative single axon, both ends of which are arrowed as “d” in **c**. White asterisks show peaks with highly correlated pixels detected manually. Scale bar, 50 µm. Bottom, correlation coefficients with seed 1 along the axonal arbor in the top panel. Blue asterisks correspond to white asterisks in the top panel. (**e**) Correlation coefficients with seed 1 (e1) and seed 2 (e2 and e3) were plotted along three putative axons. Both ends of each putative axon are arrowed as “e1”, “e2”, and “e3” shown in **c**. Blue asterisks show peaks with highly correlated pixels detected manually. “1” and “Seed 2” indicate pixels indicated by the arrowhead “1” and the arrowhead “Seed 2” in **b**, respectively. (**f**) Magnified images of the axonal boutons indicated by the seed and numbered arrowheads in **a** and **b**. Scale bar, 2 µm. The center pixels in the left-most images are seed 1 (top) and seed 2 (bottom). (**g**) Normalized Δ*F/F* traces of the axonal boutons shown in **f**. Δ*F/F* averaged over nine pixels at the center of each bouton was denoised using the CNMF algorithm. (**h**) Matrix of correlation coefficients for the activities between pairs of 12 boutons shown in **g**.

The histogram of the correlation coefficients showed a second peak around 0.6 (inset in Fig. 4g). In previous studies using TPLSM, it was reported that such highly correlated pairs of boutons were from the same axon, because boutons on the same local axon showed very similar activities^15,16^. We therefore examined whether multiple axonal structures with very similar activity patterns could be detected over the 1 mm^2^ FOV. To do so, we computed pixel-wise correlations of the calcium traces by calculating the correlations between a specific seed pixel (referred to as a ‘seed’) and all other pixels. Two examples are shown in Fig. 6a and b. In these examples, two seeds were selected manually and the pixel-wise correlation coefficients between all other pixels and the seeds were spatially mapped. The projection images of the fluorescent intensities are overlaid for display. Highly correlated pixels frequently looked like curved lines with beads, while pixels correlating with different seeds did not overlap (Fig. 6c). The density of beads with highly correlated pixels along long axon-like curves was estimated to be 68.6/mm (Fig. 6d,e). Each bead showed an axonal bouton-like structure (Fig. 6f), and the calcium transients of these pixels were similar to each other, even when the distance between some of them was ~1 mm (Fig. 6a,g). The correlation coefficients between pairs of ROIs from the same seeds were over 0.6, but those from different seeds were near to zero (Fig. 6h). These results strongly suggest that pixels that are highly correlated with the same seed presumably constitute axonal arbors with boutons from the same thalamic neuron.

## Discussion

We demonstrated that the combination of a spinning-disk unit, a variable zoom microscope, and an 8 KUHD camera allowed us to observe the activity of TC axonal boutons within a 1 mm^2^ field in a behaving mouse. We estimated that 8K-SDCLM detected 10–20% of the active boutons and ~60% of the spike events that were detected by TPLSM when the CNMF algorithm was used to extract them. These detected boutons were assumed to have large calcium transients, thereby allowing the 8K-SDCLM to be used to examine the spatial distribution of highly active boutons and their activity in a 1 mm^2^ field. However, the spatial resolution and the ability to detect boutons decrease toward the edge of the field, in which the axial FWHM was >15 µm. Especially, ROIs extracted in the area >300 µm distant from the center of the FOV need to be carefully interpreted. Axonal boutons should be sparsely labelled so as not to count multiple perpendicularly adjacent boutons as one bouton. Even with this restriction, we found that multiple axonal boutons presumably located on 1-mm-spreading axons exhibited similar neural activity. The density of active axonal boutons on single axons was estimated to be 68.6/mm. Anatomically, L1 axonal arbors from single motor thalamic neurons spread over a few millimeters in frontal cortical areas, including the primary and secondary motor areas, and the density of L1 axonal boutons was approximately 110/mm^36^. This indicates that 8K-SDCLM could detect activity in approximately 60% of the L1 axonal boutons from single thalamic neurons.

It is important to observe the activity of long-range axons in a wide field because axonal bouton activity may dynamically change along long-range axons in order to regulate information processing in the brain. Although action potentials can penetrate the end of bifurcating axonal branches, the axonal excitability can be locally modified by glial cells and secreted molecules^42–44^. The 8K-SDCLM technique could be used to test how far the effect of local glial activation on calcium transients spreads along a long axon *in vivo*. If cortical excitability differs between cortical areas, homeostasis of neural activity of TC axons spreading over these areas may be differently regulated^45^. If such axonal plasticity is related to large changes in the function of calcium channels, between-area differences in calcium transient patterns may be detected by 8K-SDCLM, even though its ability to detect a small number of action potentials was inferior to that of TPLSM. In the sensory cortex, TC axons mainly transmit their signals to layer 4, where it is difficult to resolve single axonal boutons with 8K-SDCLM. However, L1 TC axonal boutons also have important functions^46^, e.g., L1 TC axonal boutons in the visual cortex exhibit direction selectivity^47,48^. Structures of L1 TC axonal boutons in the barrel cortex are mostly stable over 1 month^6^. The 8K-SDCLM could be applied during and after sensory deprivation to examine whether and how the activity in individual L1 TC axonal boutons changes. In addition, if an infrared pulse laser with high power can be combined with the spinning device^30^ and the 8 K camera, axonal boutons in L2/3 could be imaged.

In the present study using the 8KUHD camera, only green fluorescence was used, and therefore half of the photosensors in the camera did not receive emitted light. If a red GECI were to be used, the pixels detecting red wavelengths would be able to detect its fluorescence. Simultaneous two-color imaging of red and green GECIs could therefore be used to examine long-range synaptic input patterns from different cortical and/or subcortical areas over a 1 mm^2^ field of cortical L1. Such an experiment would be very difficult to perform with any other available imaging system. In the realm of wide-field calcium imaging, OEFM was recently used to image L2/3 neuronal activity over the entire dorsal neocortex through an 8 × 8 mm cranial window at ~10 fps^20^. We showed that SDCLM with a 1 K camera allowed us to image the activity of neuronal somata at different depths of L2/3 at ~20 fps. When the pixel size is increased from 0.21 µm to ~2 µm in 8K-SDCLM, the size of the field is more than 16 × 8 mm. Thus, L2/3 neuronal activity over the entire dorsal neocortex may be imaged with a higher spatial resolution than obtainable with OEFM. The 8K-SDCLM technique has promise for examining both the cellular and synaptic activity in a wide cortical area.

In imaging of L2/3 neurons at cortical depths of >100 µm with a monochromatic 1 K camera, a red GECI was expressed to reduce scattering because scattering is lower at longer wavelengths^9^. Therefore, for imaging of L2/3 neuronal somata over the entire dorsal neocortex, a monochromic 8KUHD camera would be more suitable than the consumer product in which only a quarter of pixels would detect the red fluorescence. A substantial problem with using an 8KUHD camera involves the handling of the large quantities of data. In currently published studies using *in vivo* imaging^8–12,14^, TPLSM imaging can yield ~60 GB/hour (16 bit images of 512 × 512 pixels acquired at 30 fps), and light-sheet imaging can yield ~1.2 TB/hour^49^ (16 bit images of 1000 × 2000 × 40 pixels acquired at 2 fps). Cluster computing is effective for manipulating these large data sets^49^ and has made it possible to record imaging data at higher speeds during the image acquisitions, and to extract neuronal activities from the acquired images efficiently. However, with the 8KUHD camera, the data size could be approximately 10 TB/hour (when 16 bit images of 5310 × 4320 pixels are acquired at 60 fps), which is considerably higher than in the studies described above. If the technology of cluster computing is further improved, 8K-SDCLM has the potential to be a major imaging technique for examining information processing in the brain.

## Methods

### Animals

All animal experiments were approved by the Institutional Animal Care and Use Committee of The University of Tokyo, Japan. All experiments were performed in accordance with relevant guidelines and regulations. C57BL/6 male mice at 8–9 weeks of age were used. All mice were provided with food and water and were maintained in a 12:12 hour light-dark cycle. After attachment of a head plate to the skull as described previously^10^, mice were allowed to recover for at least 1 day before virus injections were performed.

### Virus production

rAAV1-CAG-tdTomato and rAAV1-Syn-GCaMP6s were obtained from the University of Pennsylvania Gene Therapy Program Vector Core. For imaging of R-CaMP1.07, GCaMP3 DNA of pAAV-human synapsin I promoter (hSyn)-GCaMP3-WPRE-hGH polyA^10^ was replaced with R-CaMP1.07 DNA from a pN1-R-CaMP1.07 vector construct^50^. rAAV2/1-hSyn-R-CaMP1.07 (1.3 × 10^13^ vector genomes ml^−1^) was produced with pAAV2-1 and purified as described previously^51,52^.

### Virus injection and chronic window preparation

After recovery from attachment of the head plate, mice were anesthetized with isoflurane (1–1.5%) for performance of the following surgery. For imaging of cortical cell bodies, a 3.2 mm diameter circular craniotomy (for motor cortex imaging; centered at 1.8 mm anterior to the bregma and 1.4 mm left of the midline) or a 6 × 8 mm rectangular craniotomy (for somatosensory cortex imaging; centered at the bregma) was made. Dura mater was not removed. rAAV1-Syn-R-CaMP1.07 (diluted 1/5–1/10 in aCSF; 150–200 nl each) was injected into the motor cortex (nine sites; 500 µm spacing; centered at 1.8 mm anterior to the bregma and 1.4 mm left of the midline) or somatosensory cortex (three sites; 500 µm spacing; centered at 0.5 mm posterior to the bregma and 2.5 mm left or right of the midline). The injection depth from the cortical surface was set to 300–400 µm, so that L2/3 cortical neurons would strongly express the fluorescent protein. The virus solution was injected with a Hamilton syringe and a glass micropipette, as described previously^14^. After virus injection, the craniotomy was covered with a glass window with a steel cannula (for the motor cortex) or a 6 × 8 mm rectangular coverslip (No. 2 thickness, 0.17–0.25 mm; Matsunami Glass, Osaka, Japan; for somatosensory cortex). The window with a cannula was constructed by fixing a circular glass coverslip (diameter, 3.0 mm; No. 1 thickness; 0.13–0.17 mm; Matsunami Glass) to the bottom of a circular stainless-steel cannula (outer diameter, 3.0 mm; inner diameter, 2.8 mm; height, 0.5 mm; Ohbakiko, Shizuoka, Japan) with an ultraviolet curable optical glue (NOR-61; Norland Products, NJ, USA). The craniotomy was covered directly with the coverslip, and the edges were sealed with dental resin cement (Super bond; Sun Medical, Shiga, Japan).

For imaging of TC axons, 0.2 µl of rAAV1-CAG-tdTomato or rAAV1-Syn-GCaMP6s was injected into the left thalamus (0.8–1.6 mm posterior to the bregma, 1.00–1.25 mm left of the midline, and 3.25–3.75 mm below the cortical surface, corresponding to the motor thalamic nuclei, ventral anterior/ventral lateral thalamic complex, and ventral medial nucleus^53^). The bright GECI GCaMP6s^54^ were used to label TC axons in order to increase the number of photons reaching each small pixel. rAAV1-CAG-tdTomato was diluted 1/10 in aCSF before the AAV injection. A 2.2 × 4.2 mm rectangular craniotomy was made over the left cortex (from 3.1 mm anterior to 1.1 mm posterior to the bregma and from 0–2.2 mm left of the midline, or from 1.1 mm anterior to 3.1 posterior to the bregma and from 0–2.2 mm left of the midline). Dura mater of one mouse for 8K-SDCLM imaging and two mice for TPLSM imaging was removed to increase the fluorescence signal. Dura mater of the other two mice for TPLSM imaging was intact. For imaging of 8K-SDCLM, the craniotomy was covered with a double-layer glass window^14^ constructed by joining a thin large coverslip (No. 0 thickness, 0.08–0.12 mm, 3 × 5 mm rectangular; Matsunami Glass) and a thick small coverslip (No. 4 thickness, 0.35–0.45 mm, 2 × 4 mm rectangular; Matsunami Glass) with ultraviolet curable optical glue. For TPLSM imaging, the craniotomy was covered with a triple-layer glass window constructed by joining a thin large coverslip (No. 0 thickness, 0.08–0.12 mm, 3 × 5 mm rectangular; Matsunami Glass), a thick small coverslip (No. 4 thickness, 0.13–0.17 mm, 2 × 4 mm rectangular; Matsunami Glass), and a thin small coverslip (No.1 thickness, 0.35–0.45 mm, 2 × 4 mm rectangular; Matsunami Glass) with ultraviolet curable optical glue. The craniotomy was covered directly with the coverslip, and the gap between the coverslip and the skull was filled with Vetbond (3 M, MN, USA). The edges were tightly sealed with dental resin cement.

### Imaging of fluorescent beads with SDCLM and OEFM

For SDCLM imaging, a spinning-disk unit (CSU-W1; Yokogawa Electric Corporation, Tokyo, Japan) was connected to a variable zoom microscope (Axio Zoom.V16; Carl Zeiss, Jena, Germany) with a 2.3x objective (Plan-NEOFLUAR Z 2.3x/0.57; Carl Zeiss). The zoom magnification was fixed to a value at which the total magnification of the microscope was 13x. The spinning-disk scans the whole FOV within one third of a rotation and rotates at 10,000 rpm. Thus, the maximum frame rate is 500 fps. For imaging of fluorescent microbeads (0.5 µm; Fluoresbrite Yellow Green Microspheres; Polyscience, PA, USA) around the center of the FOV (Fig. 1c,d), a CMOS camera (832 × 632 pixels; acA800-510um; Basler, Ahrensburg, Germany) was used as a photodetector and was mounted through 2 × relay optics (Yokogawa Electric Corporation). With this configuration, the pixel size at the sample plane was 0.194 × 0.194 µm. For imaging of fluorescent microbeads over a 1 mm^2^ FOV or in a tissue phantom (Fig. 1e,f,h–j), a monochromic EM-CCD camera with 1024 × 1024 pixels (iXon Ultra 888; Andor Technology, Belfast, UK) was used as a photodetector and was mounted through 1x relay optics (Yokogawa Electric Corporation). For OEFM imaging, the spinning disk was moved out of the light path of the SDCLM and an epi-illuminator module of the microscope was used to illuminate the samples. To excite the beads, a fluorescence light source (HXP 200 C; Carl Zeiss) and a filter set (38HE; Carl Zeiss; 470/40 nm excitation filter, 495 nm dichroic mirror, and 525/50 nm emission filter) were installed.

To measure the point spread function (PSF) in the SDCLM images (Fig. 1c–f), the fluorescent 0.5 µm microbeads were embedded in 0.5% low melting-point agarose (Agarose L; Nippon Gene, Tokyo, Japan) at a total concentration of 1.82 × 10^8^ beads/ml. The sample was applied on a glass bottomed dish (No. 0 thickness; Matsunami Glass) and sealed with a coverslip to avoid drying. Three-dimensional stacks of fluorescent microbead images were collected through the bottom of the inverted dish at 1 µm spacings along the Z axis. A motorized micromanipulator (MP-285; Sutter Instruments, CA, USA) was used to move the sample along the Z axis. Because of the refractive index difference between the objective immersion (air, 1.00) and the specimen (water, 1.33), the distance moved by the stage or the objective lens along the Z axis does not match with the distance moved at the actual focal plane. Thus, the latter distance was estimated to be 1.33-fold the former distance on the basis of a geometrical optics calculation^19,55^. For measuring the PSF of beads in a tissue phantom (Fig. 1g–j), a tissue phantom was prepared by mixing the 2.0 µm microbeads (Fluoresbrite Yellow Green Microspheres; Polyscience; total concentration of 2.84 × 10^7^/ml) with 0.5% low melting-point agarose and non-fluorescent polystyrene microbeads (1.0 µm; Polybead Polystyrene Microspheres; Polyscience; total concentration of 4.55 × 10^9^/ml)^33^.

### *In vivo* imaging with SDCLM, 8K-SDCLM, and OEFM

Mice were head-restrained with a head-fixing and body-holding device on a two-axis goniometer. In each imaging experiment, the angles of the goniometer were adjusted so that the glass window on the brain and the objective lens were nearly horizontal to maximize imaging quality.

For the SDCLM imaging with the 1 K camera, the monochromic EM-CCD camera with 1024 × 1024 pixels was mounted through the 1x relay optics (Yokogawa Electric Corporation). For the 8K-SDCLM imaging, the photodetector was replaced with an 8 KUHD CMOS camera (7680 × 4320 pixels; Canon, Tokyo, Japan) with 1x relay optics. The number of beams scanning the FOV and projected to the 8K camera was estimated to be 979. Images were acquired using a 488 nm diode laser (Sapphire; Coherent, CA, USA) for GCaMP6s, and a 561 nm diode laser (Cobolt Jive; Cobolt, Solna, Sweden) for R-CaMP1.07 and tdTomato. A dichroic beamsplitter (Di01-T405/488/561; Semrock, IL, USA) and barrier filters (green: 525/50, red: 617/73; Semrock) were used. The laser power under the objective was 1.9 mW for imaging of L2/3 neuronal somata and 4–5 mW for imaging of L1 axons. To obtain a wide dynamic range in the 8K-SDCLM imaging, a logarithmic conversion (WideDR; Canon) was performed on the pixel output before recording with an uncompressed 8 K video recorder (HR-7512-B; ASTRODESIGN Inc., Tokyo, Japan). The images were recorded in a 10 bit YCbCr 4:2:2 format at 60 fps. After the recording, each frame was reconverted to RGB and saved as three 16-bit monochromatic tiff images of 7680 × 4320 pixels, one for each color channel. During the 8K-SDCLM imaging shown in Fig. 4, a single head-restrained mouse performed a self-initiated lever-pull task^38^. In this task, a water-restricted mouse was trained to pull the lever with its right forelimb. When the mouse held the lever for 200 ms, the mouse was rewarded with a drop of water (4 µl) from a spout near to its mouth. To receive another reward for a lever pull, the mouse had to wait >3 s after returning the lever to its default position. The mouse was trained for five sessions (5 days). The imaging was performed 5–6 days after the implantation of the cranial windows. A program written with LabVIEW (National Instruments, TX, USA) was used to regulate the timing of the reward, and to record the lever position and licking state.

For OEFM imaging, a fluorescence light source (HXP 200 C; Carl Zeiss) and a filter set (63HE; Carl Zeiss; 572/25 nm excitation filter, 590 nm dichroic mirror, and 629/62 nm emission filter) were installed to excite tdTomato. The mouse was imaged on the day on which the craniotomy was performed.

### *In vivo* TPLSM imaging

Three mice were head-restrained under the objective 4–13 days after the implantation of the cranial windows and performed the same motor task during TPLSM imaging as was used during 8K-SDCLM imaging. Two-photon images were acquired using an FVMPE-RS system (Olympus, Tokyo, Japan) with a broadly tunable ultrafast laser (InSight DS-OL; Spectra Physics, CA, USA) tuned to 940 nm. The back aperture of the objective (XLPLN25XWMP2; back aperture diameter, 15.1 mm; numerical aperture [NA], 1.05; Olympus) was underfilled with the diameter-shortened (7.2 mm) laser beam to reduce the effective excitation NA of the objective^14^. The dimensions of the FOV were 512 × 512 pixels (127.2 × 127.2 µm) for all imaging fields. The imaging fields were at 40–60 µm depth below the cortical surface. The laser power was adjusted to maintain a relatively constant fluorescent intensity from the axonal boutons (5.94–11.9 mW). A series of 10,800 continuous images was acquired at 30 Hz. A 570 nm dichroic mirror (FV30-FGR; Olympus) and a bandpass filter at 495–540 nm were used. For XYZ imaging (Fig. 5i), three planes with an interval of 8 µm in the same horizontal field were sequentially imaged in three mice. For each plane, the dimensions of the FOV were 512 × 256 pixels (84.9 × 42.4 µm) and a series of 1860 or 2230 images was acquired at 6.2 Hz.

### Image processing

Analyses were performed using MATLAB (2015b or 2016a; Mathworks, MA, USA) and ImageJ (National Institute of Health, MD, USA). In the analysis of images obtained with 8K-SDCLM, only the image from the green channel was used. Lateral displacements of acquired image sequences were corrected using TurboReg^40^ for imaging with SDCLM, 8K-SDCLM and XYZ imaging with TPLSM, and a non-rigid motion correction algorithm^56^ for XY imaging with TPLSM. We refer to the images after motion correction as ‘motion-corrected raw images’. A CNMF^34^ algorithm was used to determine ROIs. This algorithm extracts a time series of a calcium trace (temporal component) with its spatial position and shape (spatial component) by minimizing a residual between the motion-corrected raw image sequence and the reconstructed image sequence generated by multiplying the spatial and temporal components. In the analysis of the neuronal somata in SDCLM images and the synaptic boutons in XYZ images with TPLSM, the spatial component and fluorescence signals in the motion-corrected raw images were multiplied at each pixel and then averaged over pixels within each ROI, to determine the fluorescence signals for each ROI. To calculate the Δ*F/F*, the baseline fluorescence intensity (*F*) was estimated as the eight percentile values over an interval of ±15 s around each sample time point^10^ in the analysis of the neuronal somata in SDCLM images. In the analysis of XYZ imaging, the baseline (*F*) was estimated as the eight percentile values over the entire imaging period (1860 or 2230 frames, corresponding to 5 or 6 minutes).

As the signal-to-noise ratio of 8K-SDCLM images was lower than that of TPLSM and SDCLM, the 8K-SDCLM images were filtered with a Gaussian kernel. Both XY images acquired by the 8K-SDCLM and TPLSM were resampled to a frame rate of 6 Hz before application of the CNMF algorithm. In the analysis of the synaptic boutons in 8K-SDCLM and TPLSM imaging, we assumed that the fluorescence signals obtained by 8K-SDCLM and TPLSM would reflect similar neuronal activities during the similar behaviors. The fluorescence of each ROI (*F*_*roi*_) was calculated by multiplying the spatial components of CNMF and the motion-corrected raw images. To remove neuropil fluorescence from *F*_*roi*_, an iteratively re-weighted least-squares algorithm (*robustfit* in MATLAB) that discounts large deviations from the fitted linear relationship was used^41^. For each ROI, the mean fluorescence of a 2 µm neighborhood was defined as *F*_*neuropil*_. Using all the time points except for those where *F*_*roi*_ values exceeded the time-averaged mean plus 2S.D. in the 8K-SDCLM image, or 1S.D. in that of TPLSM, a robust linear regression between *F*_*roi*_ and *F*_*neuropil*_ values was calculated, and its slope was used to define a contamination factor^41^. In each imaging field, the median value of the contamination factors of the ROIs was defined as *r*. Then, for each ROI, *F*_*roi*_ *– r* × *F*_*neuropil*_ was used to define *F*_*true*_, the estimated value of the fluorescence without neuropil contamination. The acquired calcium trace was denoised using the algorithm in CNMF. For the images obtained with the 8K-SDCLM, the calculation of Δ*F/F* followed the description above. For the images obtained with the TPLSM, the baseline (*F*) was estimated as the eight percentile values over the entire imaging period (10,800 frames, corresponding to 6 minutes).

### Comparisons of detected ROIs and spike events between TPLSM and 8K-SDCLM images

When ROI sizes were compared for Fig. 4c,d, the ROIs in the images obtained with TPLSM (1891 ROIs from six imaging fields) and 8K-SDCLM (395 ROIs from three 213 × 213 µm areas, Fig. 4a) were used. ROIs that were within ~4 µm of the edge of the imaging fields (238/1891 ROIs in the TPLSM images and 22/395 ROIs in the 8K-SDCLM images) were excluded. To compare the ROI sizes, the ROI centers were defined by the coordinates where the value of the spatial component of each ROI was at a maximum, and the spatial component was normalized to this maximum value.

The distribution of the fluorescence signals obtained with 8K-SDCLM was determined for 100 randomly sampled pixels within a rectangle field, as shown in Fig. 4ai. For these pixels, the fluorescence signals in the motion-corrected raw images were divided by the variance of the time-averaged fluorescence signals calculated over all the pixels in the rectangle field except for those pixels that appeared to correspond to blood vessels (normalized fluorescence signals). The distribution of the normalized fluorescence signals was fitted to a gamma distribution and the parameters were then estimated (shape parameter [*k*] of 2.57 ± 0.02 and scale parameter [*θ*] of 2.88 ± 0.01, *n* = 100). The time-averaged normalized fluorescence signal per pixel was linearly proportional to the variance of the corresponding gamma distribution (*k* × *θ*^2^). The slope of the variance against the time-averaged normalized fluorescence signal was defined as the *NR*. Then, for each pixel at each time point in the imaging data acquired with TPLSM (six fields), the random numbers from the gamma distribution with *k* of 2.57 and *θ* = √(*NR* × the time-averaged normalized fluorescence/*k*) were added (*NR* = 1, 2, 3, 3.75, and 5). ROIs and their spike events were extracted from a time series of these images using the CNMF algorithm. In this simulation without the pair-wise correlation analysis, the procedure to remove neuropil fluorescence was not performed.

The XYZ imaging data were used to estimate the overlap of ROIs along the Z axis. For each plane, ROIs were extracted with the CNMF algorithm, and the overlaps between pairs of ROIs on 8 µm-apart planes were calculated using their ROI areas. The ROI areas were defined as areas where the normalized spatial component exceeded 0.5, consistent with the definition of the ROI size. When the overlapping area exceeded 50% of the ROI area, this pair was regarded as an overlapping one, where it could be difficult to separately detect the two ROIs in separate planes. If overlapping ROIs showed a pair-wise correlation coefficient >0.6 in the temporal component, the pair was assumed to be from boutons on the same axons or the same bouton and was removed from the estimation (20 ROIs from three horizontal locations from three mice). The 8-µm Z interval was used because nearer planes had more overlapping ROIs with a pair-wise correlation coefficient >0.6, which could be the same boutons.

### Pixel-wise correlation of the axonal bouton activity over the FOV

Time series data from 10,000 frames were used, corresponding to a 170 s imaging period. To improve the signal-to-noise ratio, the image size of 5310 × 4320 pixels was down-sampled to a size of 2655 × 2160 pixels and the trace time series of each pixel were averaged over 10 frames. Thus, pixel-wise correlations between pairs of 1000 time point vectors were calculated for each of the 2655 × 2160 pixel locations. Two example seeds that mapped highly correlated pixels to an axon-like structure were selected.

The number of beads with highly correlated pixels on four putative axonal arbors shown in Fig. 6c was manually counted. The value (144 boutons/2.10 mm) was similar to 150 boutons/2.1 mm obtained with *findpeaks* in Matlab.

### Data availability

The data that support the findings of this study are available from the corresponding author upon reasonable request.

### Code availability

The MATLAB codes for the CNMF algorithm^34^ and the non-rigid motion correction algorithm^56^ are available on the code author’s GitHub repository (https://github.com/epnev/). The ImageJ plugin for the TurboReg algorithm^40^ is available on its author’s website (http://bigwww.epfl.ch/thevenaz/turboreg/). Other codes for analyses are available from the corresponding author upon reasonable request.

## Electronic supplementary material

Supplementary Information

Supplementary Video 1

Supplementary Video 2
