# Supplementary material for: In vivo wide-field calcium imaging of mouse thalamocortical synapses with an 8 K ultra-high-definition camera
Source: Sci Rep. 2018 May 29;8:8324. doi: 10.1038/s41598-018-26566-3 (PMC5974322; doi:10.1038/s41598-018-26566-3)
Supplement: Supplementary file 1 — Supplementary Information [file 41598_2018_26566_MOESM1_ESM.pdf]

Supplementary information

In vivo wide-field calcium imaging of mouse thalamocortical synapses with an 8K ultra-high-definition camera

Eriko Yoshida, Shin-Ichiro Terada, Yasuyo H. Tanaka, Kenta Kobayashi, Masamichi Ohkura, Junichi Nakai, and Masanori Matsuzaki

This file includes

- Legends of Supplementary Video

Supplementary Video 1 | *In vivo* SDCLM imaging of R-CaMP1.07-expressing L2/3 neurons.

SDCLM imaging of L2/3 neuronal somata in the motor cortex that expressed R-CaMP1.07. The depth was 120 µm from the cortical surface. The FOV size is 1080 × 1080 µm. The time series of the images acquired at 20 fps is averaged over 10 frames. The movie plays at 10× the true speed.

Supplementary Video 2 | Imaging of thalamocortical axonal boutons with 8K-SDCLM.

8K-SDCLM imaging of GCaMP6s-expressing TC axons. The left panel shows the image, which is down-sampled to a size of 2655 × 2160 pixels and filtered with a Gaussian kernel. The FOV size is 1106 × 900 µm. The right panels are magnified images of the three white boxes in the left panel. Each FOV size is 185 × 185 µm. The time series of all images acquired at 60 fps were averaged over 40 frames. All movies play at 10× the true speed.
